# Phylogenetic Relationships and Evolution of the Androecia in Ruteae (Rutaceae)

**DOI:** 10.1371/journal.pone.0137190

**Published:** 2015-09-02

**Authors:** Lai Wei, Xiao-guo Xiang, Yin-zheng Wang, Zhen-yu Li

**Affiliations:** 1 College of Life Sciences, Beijing Normal University, Xinjiekouwai Street, Beijing, China; 2 State Key Laboratory of Systematic and Evolutionary Botany, Institute of Botany, Chinese Academy of Sciences, Xiangshan, Beijing, China; Royal Botanic Gardens, Kew, UNITED KINGDOM

## Abstract

*Ruta*, which belongs to tribe Ruteae, is the type genus of the subfamily Rutoideae and the family Rutaceae. Molecular systematic studies have shown that the genera in Ruteae are closer related to Aurantioideae than to most other genera of Rutoideae, some of the genera traditionally placed in Ruteae have been shown to be nested within the Aurantioideae clade, but the diagnostic characters for determining new patterns in the relationship are poor. In this study, we investigated the floral development of *Boenninghausenia* in Ruteae (*sensu stricto*), *Haplophyllum* in the basal position of Aurantioideae and *Murraya* in traditional Aurantioideae using scanning electron microscopy. The androecium of *Boenninghausenia* is obdiplostemony. As androecia in other genera within Ruteae (s.s.) are also obdiplostemonous, reconstruction of the ancestral state indicates that obdiplostemony is an ancestral character in this clade. Because the androecia of *Haplophyllum* and *Murraya* are also obdiplostemonous, obdiplostemony is also an ancestral character in Aurantioideae clade. The ancestral state reconstruction indicates this character can serve as a synapomorphy of the Ruteae-Aurantioideae clade. The results of our work also shed light on the evolution of the androecium in Rutaceae, as the obdiplostemony of this group is clearly derived from haplostemony in the ancestral genera in Rutaceae and has develop into polyandry by increasing antepetalous stamens.

## Introduction

**Fig 1:**
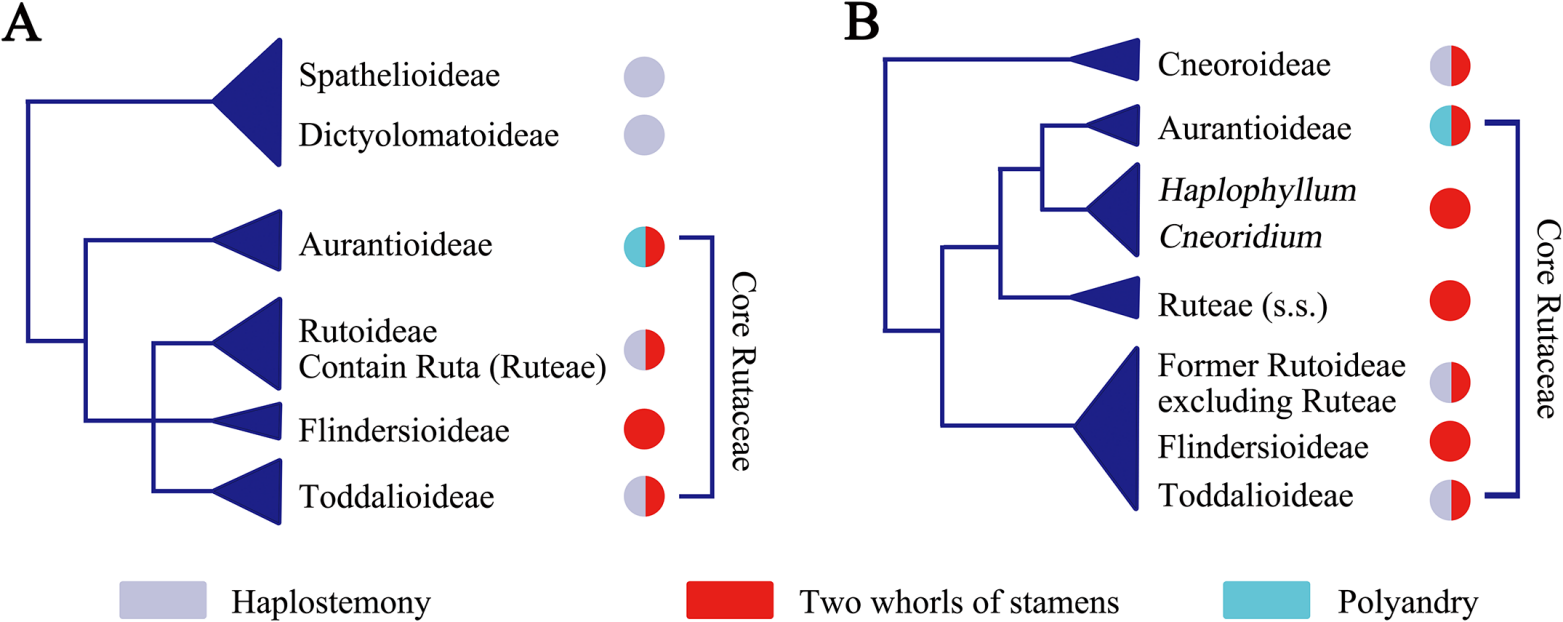
Comparing the traditional system of Rutaceae with current molecular phylogeny with general androecium characters mapping in the tree: A: Traditional system of Rutaceae (mostly based on Engler’s treatment [6,7]). B: Summary of the major clades of Rutaceae based on published molecular phylogenies [2,4,11,14,17].

The Rutaceae family, which is well known due to its economic importance (*Citrus*), is a large family including 154 genera and approximately 2100 species [1]. The distribution of this family is nearly cosmopolitan, with species in both the Old World and New World, but it is mainly tropical and subtropical [2–5]. An important systematic treatment of the group was conducted by Engler [6,7], who recognized seven subfamilies. Along with the modern systematics of this family, many of the subfamilies are controversial [8,9]. Recently, great progress has been made in the infra-familial phylogeny through molecular systematic studies [2–5,10–14]. The consensus consists of traditional subfamilies that are not natural groups, and Rutoideae, Toddalioideae, Flindersioideae and Aurantioideae form a ‘core Rutaceae’ clade [1]. At the base of ‘core Rutaceae’, former member of Spathelioideae and Dictyolomatoideae formed a clade under the name of Cneoroideae (subfamily Cneoroideae), sister to ‘core Rutaceae’. This clade is clearly the most early-diverging within Rutaceae [15,16]. In the core Rutaceae, Rutoideae and Toddalioideae are merged together, whereas the genera within Flindersioideae are dispersed in both of the clades. Under this systematic framework, the position of *Ruta* seems unusual (Fig 1). As the type genus of Rutoideae, *Ruta* is closely linked to Aurantioideae rather than Rutoideae [2,4,13,17].

*Ruta* and related genera are grouped in the tribe Ruteae. The Englerian tribe Ruteae was mainly defined based on genera with the same life form, habits and distributions. Recent molecular phylogenetic studies have shown that Ruteae has a closer relationship with Aurantioideae than with other Rutoideae groups [13,14,17]. If this relationship is accurate, it may result in important changes in subfamily nomenclature. Despite the definitive molecular systematics results, the morphological foundation for new patterns of relationships is currently poor. Although Ruteae is noteworthy for the different growth habits of its members compared with the other genera within Rutoideae, diagnostic characters are lacking regarding its affinity with Aurantioideae.

It is no exaggeration to state that morphological data, based on the external form of organisms, have been and still are the most commonly used type of data in plant classification [18]. Among such data, the androecium of flowers is obviously of high taxonomic value within angiosperms, and the anthers exhibit many different sizes, shapes, and numbers [19]. The family Rutaceae bears varied types of androecia, with haplostemonous, diplostemonous, obdiplostemonous and polyandrous androecia present in different genera [1,20]. The androecia of Ruteae are heterogeneous among the Old World Rutoideae, exhibiting two whorls of stamens. However, the existence of two whorls of stamens is not rare in Rutoideae, whose members are mostly distributed in Africa, Australia and South America. All except one genus (*Harrisonia*) of the early-diverging subfamily Cneoroideae are haplostemonous.[1]. Meanwhile, in the genera of Old World Rutoideae, there are some other genera with haplostemonous androecia, such as *Zanthoxylum*, *Phellodendron*, *Tetradium* and *Toddalia*. These genera represent the extant genera in ‘core Rutaceae’ with the earliest fossil record [21]. Therefore, determining the evolutionary trends involving these androecia will be useful for clarifying the relationships among the different genera.

In past decades, developmental data have been used taxonomically to some extent as a corollary to comparisons of mature floral structures [22–25]. This method applies to revealing homologies among mature structures [26]. Stebbins [27,28] and Endress [23] have rightly noted the great value of developmental data for interpreting evolutionary relationships and trends at higher levels of the hierarchy in angiosperms. Many studies have examined floral development and make contributions of systematic relevance for different taxa [29–34]. Within Ruteae, the floral development of *Psilopeganum sinense* and *Ruta graveolens* has been studied in detail [35]. Both genera possess an obdiplostemonous androecium. Compared with the haplostemonous androecium found in *Phellodendron* [36] and *Tetradium* [37], obdiplostemony in these genera is a derived character [33]. Aurantioideae also includes genera with two whorls of stamens, such as *Clausena* and *Murraya*. According to current molecular systematics, these genera represent the ancestral clade in Aurantioideae [38]. Therefore, determining the mode of androecium development in these genera is important for understanding the relationship between Ruteae and Aurantioideae.

There are seven genera within Ruteae: *Ruta*, *Psilopeganum*, *Haplophyllum*, *Boenninghausenia*, *Dictamnus*, *Thamnosma* and *Cneoridium*. Recent phylogenetic studies showed that Ruteae is not monophyletic [39,40]. *Ruta*, *Psilopeganum*, *Thamnosma* and *Boenninghausenia* form a clade [17] that we name Ruteae *sensu stricto* here. The other two genera, *Haplophyllum* and *Cneoridium*, are placed in the basal position of the Aurantioideae clade [39,40]. Recently, *Dictamnus* was excluded from Ruteae based on chemosystematics, anatomy and molecular systematics [13,39–41], and the floral development of *Ruta* and *Psilopeganum* has been studied. Therefore, we chose *Boenninghausenia albiflora* to represent the Ruteae (s.s.); *Haplophyllum dauricum* to represent the basal Aurantioideae group and *Murraya exotica* to represent the genera in traditional Aurantioideae with two whorls of stamens. The floral developments of these genera were studied. The aims of this study were to clarify the mode of androecium development in these genera through floral ontogeny; to reconstruct the ancestral character of the androecium in Ruteae and discuss the evolutionary trend of the androecium in Rutaceae; and finally, to clarify the relationships between Ruteae and Aurantioideae.

## Materials and Methods

### Taxon Sampling

*Haplophyllum dauricum* plants were collected from the Inner Mongolia Grassland Ecosystem Research Station (Wei 10117) of the Chinese Academy of Sciences. *Boenninghausenia albiflora* plants were collected on West Mountain in Kunming (Wei 12097). *Murraya paniculata* plants were collected from the Beijing Botanic Garden (Wei 10023), Chinese Academy of Sciences. Voucher specimens were deposited in the Chinese National Herbarium (PE).

### Floral Development Analysis

The plant material was fixed in formalin—acetic acid—alcohol (FAA). The FAA-fixed materials were dehydrated in an ethanol series. Flower buds were dissected and examined in 95% ethanol using a dissecting microscope, then transferred through an ethanol-iso-amyl acetate series, critical-point dried, mounted on a metal stub and sputter-coated with gold/palladium. The flower buds were observed, and micrographs taken with a Hitachi S-4800 scanning electron microscope (SEM) (Hitachi, Tokyo, Japan) at 10 kV.

### Ancestral State Reconstruction

To trace the evolutionary history of the androecium, we conducted character-state reconstruction in Mesquite v2.75 (http://mesquiteproject.org) [42]. The topology of the sampled species was determined according to Groppo et al. [4], Salvo et al. [13], Poon et al. [11], Morton et al. [17], and Manafzadeh et al. [43]. According to androecium state of species, we coded four states: 1 for haplostemony, 2 for diplostemony, 3 for obdiplostemony and 4 for polyandry. Because of no androecium data of *Thamnosma* and *Cneoridium*, we coded them as missing. The evolution of the androecium was reconstructed using a maximum likelihood approach with the Markov k-state one-parameter (MK1) model.

## Results

### Floral Initiation and Development in *Haplophyllum dauricum*

**Fig 2:**
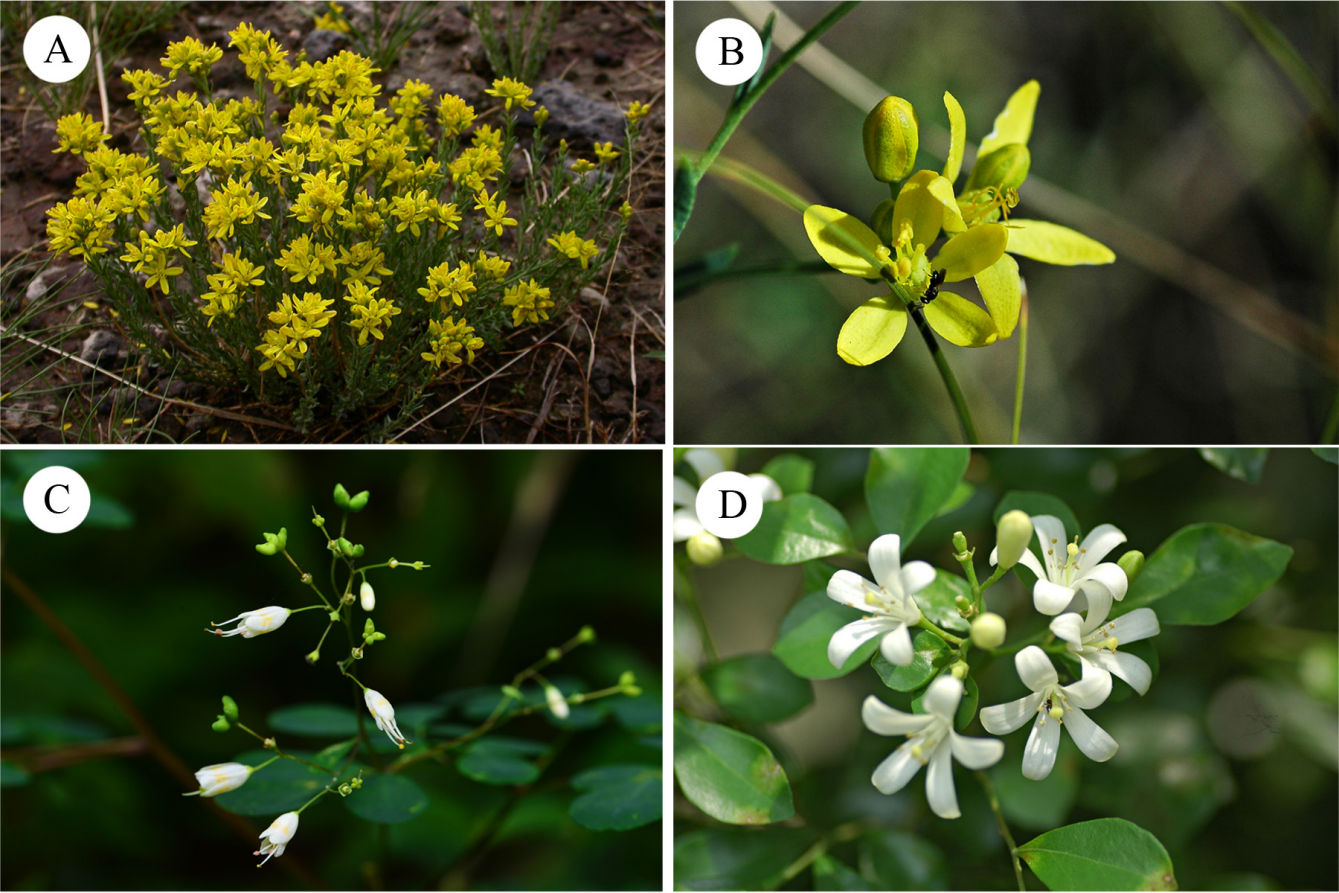
*Haplophyllum dauricum* (A, B), *Boenninghausenia albiflora* (C) and *Murraya exotica* (D).

The actinomorphic flowers are grouped in loose cymes. The flowers are usually pentamerous, with ten stamens arranged in two whorls. In the mature flower, the 10 stamens surround the gynoecium (Fig 2B). The gynoecium is usually 3-carpellate, but the merosity ranges from two to five.

**Fig 3:**
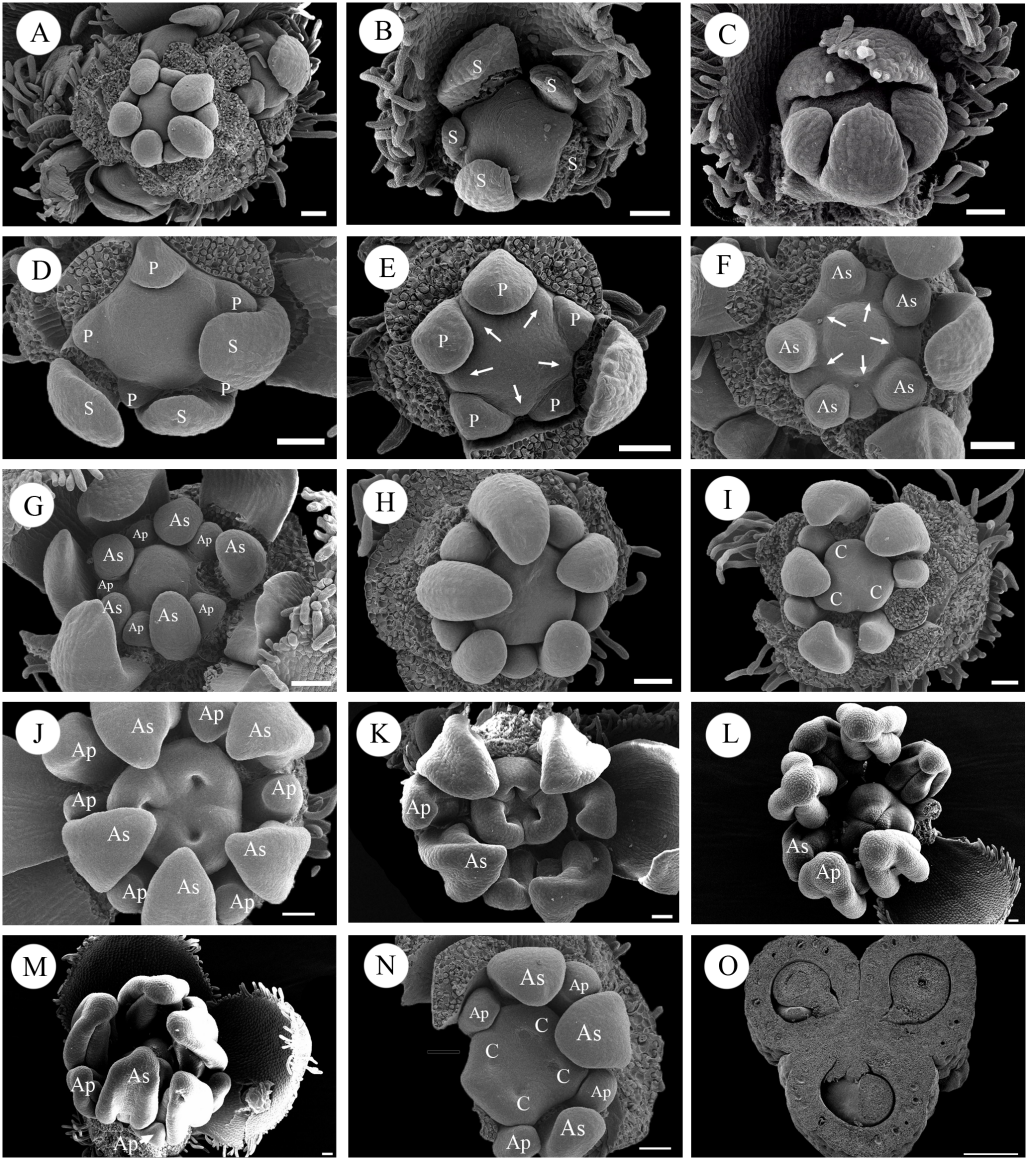
Floral development in *Haplophyllum dauricum*. A: Apical view of a young inflorescence with lateral subunits. B, C: Apical view of a young floral bud showing the initiation sequence of the sepals. D: Initiation of petals. E: Initiation of antesepalous stamens. Arrows indicate the primordia. F: Initiation of antepetalous stamens. Arrows indicate the primordia. G: Fully initiated androecium showing the different sizes of antesepalous and antepetalous stamens. H: Apical view of a floral bud in the middle stage. I: Initiation of carpels. J: Development of the three carpels (note: in rare cases, as shown here, an additional stamen may be formed). K: Later stage of carpel development. L: Post-genital union of three carpels. M: Later stage of the floral bud, showing the peripheral position of the antepetalous stamens. N: Apical view of a floral bud with a tetramerous mutation showing the antepetalous position of the carpels. O: Transection of the mature ovary. Abbreviations: S, sepal; P, petal; As, antesepalous stamen; Ap, antepetalous stamen; C, carpel. Scale bars = 40 μm.

The flower primordia and related bracts and bracteoles arise helically and acropetally on the main inflorescence branch (Fig 3A). The bract and bracteoles are easily distinguished from the sepals and petals by the trichomes along their margins (Fig 3B). The first sepal primordium emerges on the abaxial side of the flower after the two lateral bracteoles become visible, followed by others in rapid sequence (Fig 3B). The calyx is initiated in a classical quincuncial spiral sequence (Fig 3C). During the development of the calyx, the floral apex increases in height and flattens, and the five petal primordia appear in the antesepalous position in very close succession (Fig 3D). Initiation may begin on the abaxial side, as the primordia are slightly larger in the abaxial area.

Immediately after initiation of the petals, the stamen primordia begin to emerge. The first whorl of the stamen primordia arises inside the petal whorl in an antesepalous position (arrows in Fig 3E). The antepetalous stamens arise acropetally after the antesepalous stamens (arrows in Fig 3F). At this stage, the development of the androecium is diplostemonous (Fig 3G). The antesepalous stamens grow rapidly, whereas the growth of the antepetalous stamens is retarded. As a result, the two whorls of stamens are clearly different in size (Fig 3H). In late development, each stamen enlarges laterally and differentiates into an anther and filament and the median and transverse furrows divide the anther into two thecae and four pollen sacs. After stamen initiation, the floral apex becomes flat and the carpel primordia emerge around its margin (Fig 3H). As the antesepalous stamens grow rapidly and occupy more space on the floral apex, the three carpel primordia appear in an antepetalous position (Fig 3I). Three furrows become visible, and the carpels soon rise above the floral apex (Fig 3J). Along with the development of the carpels, the antepetalous stamens shift to the periphery (Fig 3K–3M). In the mature flower, the carpels become united (Fig 3L) and further differentiate to form the style and stigma. In some rare flower buds with mutations, while all of the floral organs are isomerous and tetramerous (flowers with four carpels and eight stamens in two whorls), the developmental succession is mostly the same, with the carpels clearly arising in the antepetalous position (Fig 3N).

### Floral Initiation and Development in *Boenninghausenia albiflora*

The actinomorphic flowers of *Boenninghausenia albiflora* are grouped in monochasium. The floral merism is strictly tetramerous (Fig 2C). The eight stamens are grouped into two whorls, and the antesepalous stamens are longer than the antepetalous stamens.

**Fig 4:**
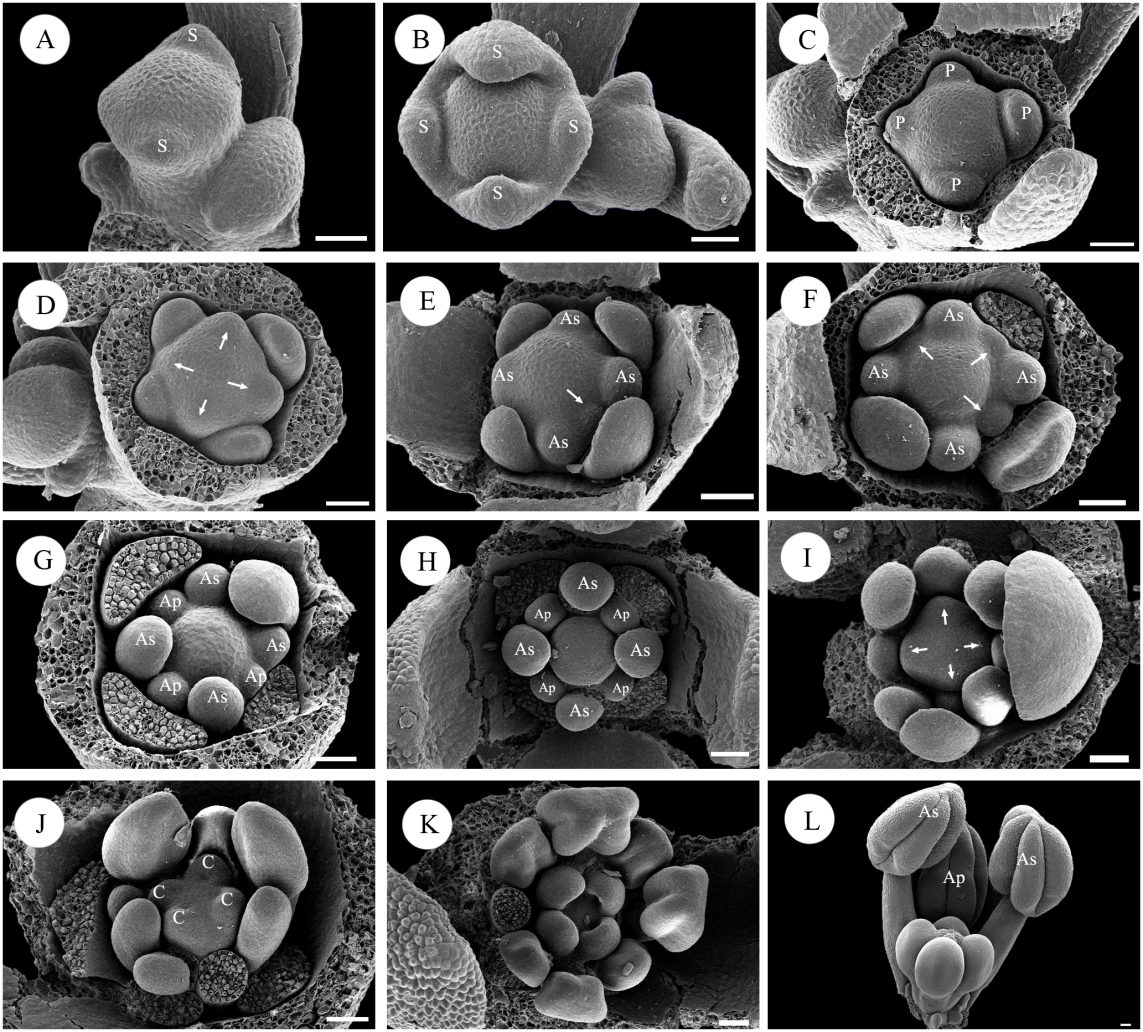
Floral development in *Boenninghausenia albiflora*. A: Apical view of a young flower located terminally on the inflorescence apex. B: Initiation of sepals. C: Initiation of petals. The different petal sizes indicate the initiation sequence. D: Initiation of antesepalous stamens. Arrows indicate the primordia. E, F: Sequential initiation of antepetalous stamens. Arrows indicate the primordia. G: Fully initiated androecium showing different sizes between antesepalous and antepetalous stamens. H: Apical view of a floral bud in the middle stage. I: Initiation of carpels. Arrows indicate the primordia. J: Development of the four carpels at the antepetalous position. K: Later stage of carpel development. L: Part of a nearly mature flower showing the position and different lengths between antesepalous and antepetalous stamens. Abbreviations: S, sepal; P, petal; As, antesepalous stamen; Ap, antepetalous stamen; C, carpel. Scale bars = 40μm.

The inflorescence apex becomes a floral primordium, and two bracteoles arise on the lateral side (Fig 4A). The secondary floral primordium usually arises on the axil of the bracteoles (Fig 4B). The sepal primordia occur in a ring, consisting of the four sepal lobes. The four sepal lobes arise out of the ring in a later stage, and the adaxial and abaxial lobes are slightly larger than the lateral lobes (Fig 4B). After the appearance of the sepals, the petal primordia begin to arise. The first petal primordium usually occurs on the abaxial side, and the other three petal primordia arise in very close succession either clockwise or counter-clockwise (Fig 4C).

The development of the androecium is nearly the same as in *Haplophyllum dauricum*. Two whorls of stamens arise in a regular order, with the antesepalous stamens arising first (arrows in Fig 4D). The antepetalous stamens arise at the same level as the antesepalous stamens (arrows in Fig 4E and 4F). The antesepalous stamens are larger than the antepetalous stamens throughout development (Fig 4G). In late development, each stamen differentiates into an anther and filament. After stamen initiation, the floral apex becomes flat, with four corners (Fig 4H). Four independent carpel primordia emerge simultaneously at the antepetalous positions (Fig 4I and 4J). The four carpels become post-genitally united and further differentiate to form the style and stigma (Fig 4K and 4L).

### Floral Initiation and Development in *Murraya exotica*

The actinomorphic flowers of *Murraya exotica* are grouped in dichasial cymes. The flowers are pentamerous, and the gynoecium is composed of two carpels. The ten stamens, of unequal length, are arranged in two whorls and surround the gynoecium (Fig 2D).

**Fig 5:**
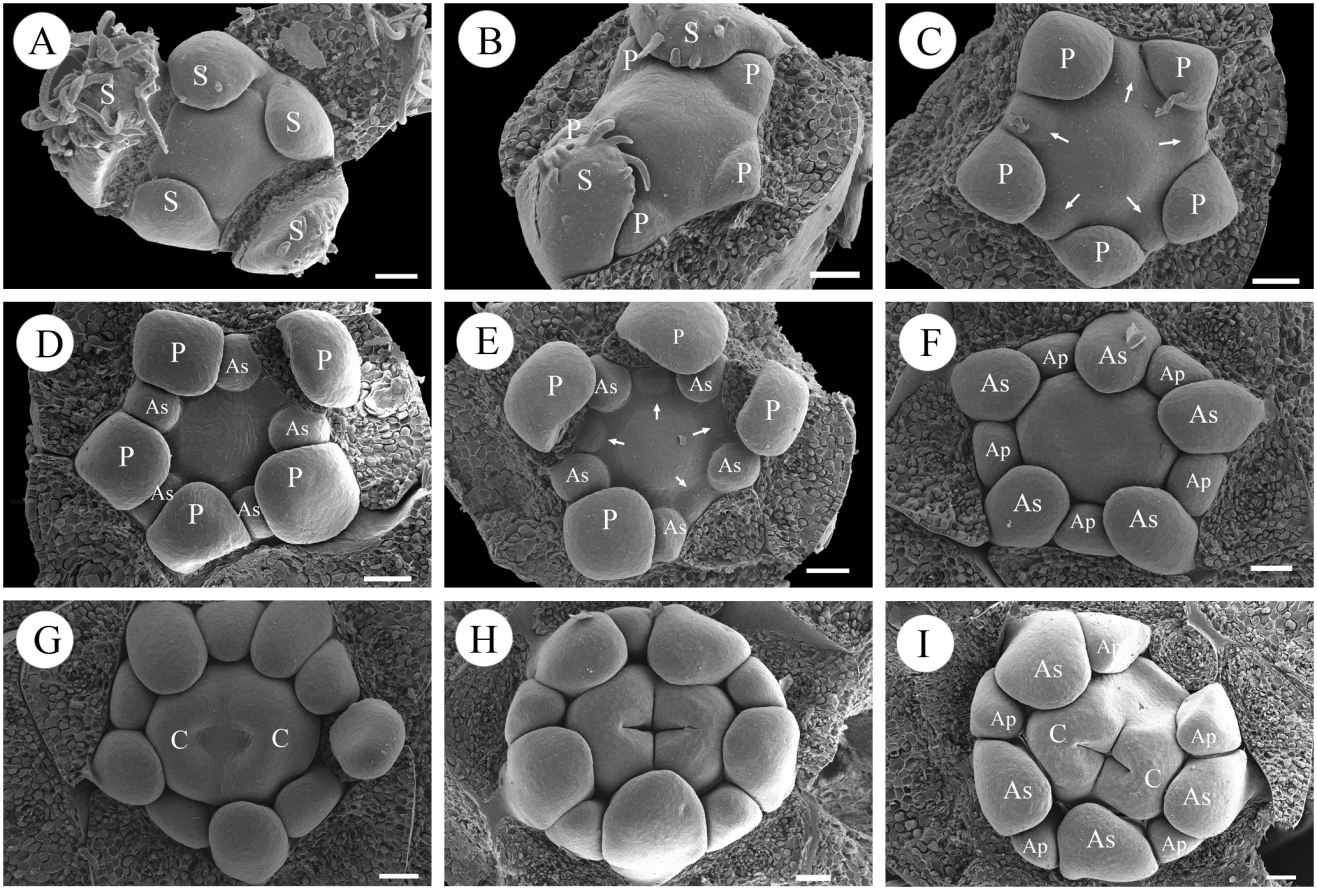
Floral development in *Murraya exotica*. A: Apical view of a young floral bud showing the initiation sequence of the sepals. B: Initiation of petals. C: Initiation of antesepalous stamens. Arrows indicate the primordia. D: Fully initiated antesepalous stamens. E: Initiation of antepetalous stamens. Arrows indicate the primordia. F: Fully initiated androecium showing the different sizes of antesepalous and antepetalous stamens. G: Initiation of carpels. H: Development of the two carpels. I: Apical view of a floral bud in the late stage. Abbreviations: S, sepal; P, petal; As, antesepalous stamen; Ap, antepetalous stamen; C, carpel. Scale bars = 40 μm.

Floral development is similar to that of *Haplophyllum dauricum* and *Boenninghausenia albiflora*. The floral primordia are subtended by their respective bracts. The sepal primordia begin to emerge after two bracteoles become visible. The first sepal primordium emerges on the abaxial side, followed by the adaxial and lateral sepals (Fig 5A). The initiation of the calyx follows a classical 2/5 quincuncial spiral sequence. The surface of the sepals is covered by trichomes (Fig 5A). The development of the petals begins on the abaxial side in very close succession immediately after sepal initiation (Fig 5B).

The development of the androecium is nearly the same as in *Haplophyllum dauricum* and *Boenninghausenia albiflora*. The ten stamens divide into two whorls, and the antesepalous stamens appear first (arrows in Fig 5C), followed by the antepetalous stamens (arrows in Fig 5E). The two whorls of stamens emerge acropetally and appear diplostemonous (Fig 5F). The two whorls of stamens are different in size, reflecting their different growth rates (Fig 5F). In late development, each stamen differentiates into an anther and filament. After stamen initiation, the floral apex begins to elongate and two furrows, representing two carpels, emerge at the lateral position (Fig 5G). The carpels become basally fused during enlargement, forming a continuous structure (Fig 5H). At a later stage, the carpellary locules begin to inflate, and as the space is occupied by the antesepalous stamens, this inflation causes the antepetalous stamens to shift to the periphery (Fig 5I), such that in the mature flower, the antepetalous stamens are outside the antesepalous stamens.

### Ancestral State Reconstruction

**Fig 6:**
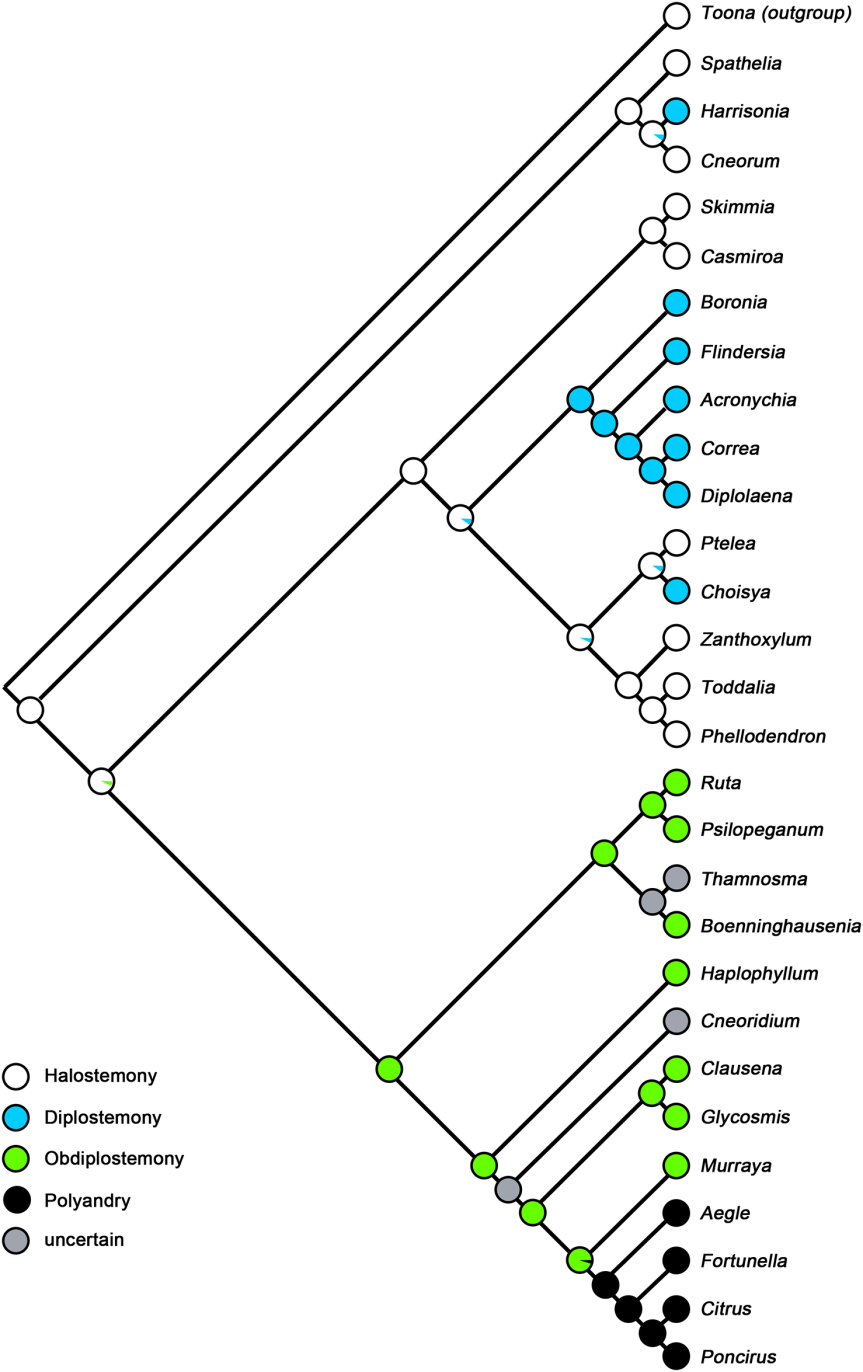
Ancestral state reconstruction for the androecium in the major clade Rutaceae. The topology of the sampled species was determined according to Groppo et al. [4], Salvo et al. [13], Poon et al. [11], Morton et al. [17], and Manafzadeh et al. [43]. The different colors represent the different status of androecium.

The ancestral state reconstruction (Fig 6), based on 28 out of 154 genera of Rutaceae, but sampling all major lineages, clearly showed that haplostemony is ancestral in Rutaceae as the genera in early-diverging clade Cneoroideae mostly have haplostemonous androecia except *Harrisonia* [1].

The core Rutaceae is divided into two main clades: the Ruteae-Aurantioideae clade and a clade consisting of the remaining genera belonging to the former Flindersioideae, Toddalioideae and Rutoideae (= Amyridoideae sensu Morton & Telmer [17]). In the Toddalioideae-Rutoideae clade, the ancestral state is most likely haplostemony with diplostemony having evolved several times separately, especially in the genera distributed in the New World, such as *Correa*, *Acronychia* and *Choisya*.

In the Ruteae-Aurantioideae clade, the ancestral state is obdiplostemony. The genera in Ruteae and the ancestral genera in Aurantioideae, such as *Clausena*, *Glycosmis* and *Murraya* all have obdiplostemonous androecia. Comparing to these genera, the remaining genera in the clade such as *Citrus*, *Poncirus* and *Fortunella* all have polyandrous androecia, making polyandry secondary in this clade.

## Discussion

The mode of androecium development in *Boenninghausenia albiflora* is clearly obdiplostemony. Although the two whorls of stamens arise in a regular order and are approximately inserted in one whorl, the carpels arise at the antepetalous position. This pattern may correspond to the maximum use of space and is a clearly an indication of obdiplostemony [44,45].

In the early stage of the floral ontogeny of *Haplophyllum dauricum*, the arrangement of the stamen primordia is in accord with Hofmeister’s rule as well as the inhibitory field theory. That means the new lay-out should arise with the least possible disturbance of neighboring structures [46]. In later stages, because the antesepalous stamens grow rapidly and occupy more space on the floral apex, the carpel primordia are initiated at the antepetalous position. Along with the inflation of the carpellary locules, the antepetalous stamens shift externally. It is worth noting that in some rare buds with a tetramerous mutation, while the flower is isomerous, the carpel arises at the antepetalous position. Therefore, the androecium in *H*. *dauricum* is also obdiplostemonous.

In the typical developmental pattern of obdiplostemony, the abnormal placement of two whorls of stamens is mainly caused by the irregular inception of certain floral organs, e.g., the centrifugal inception of the antepetalous stamens or the early inception of the carpels [20,47]. Compared with obdiplostemony, the developmental sequences of *Boenninghausenia albiflora* and *Haplophyllum dauricum* are regular. Only after inception in the acropetal sequence are the antepetalous stamens pushed outward by the growing carpels. This pattern corresponds to secondary obdiplostemony according to Ronse De Craene [20], indicating that obdiplostemony was a late phenomenon in these taxa [48].

Knowledge of ontogenetic patterns generally contributes to systematics and phylogenetic considerations [48], but previous studies have indicated that obdiplostemony is not relevant for systematics [49] because this phenomenon can be found in most members of the Rosids with pentamerous isomerous flowers, such as Zygophyllaceae, Oxalidaceae, Onagraceae and Tiliaceae [20,44,45,50]. As these families belong to different clades of Rosids, obdiplostemony is thought to have evolved separately. However, at the infrafamilial level, the situation may be different. In Rutaceae, the most early-diverging lineage Cneoroideae is represented by haplostemony [1]. Most genera in this subfamily have small haplostemonous flowers like *Cneorum* [51]. The ancestral state reconstruction also showed that haplostemony is the ancestral character in Rutaceae. The Toddalioideae-Rutoideae clade also contains some genera that are haplostemonous, such as *Zanthoxylum*, *Toddalia* and *Phellodendron*. Intrestingly, these genera represent the earliest fossil record of the extant genera in Rutaceae [21]. So, compared with haplostemony, two whorls of stamens is a derived character. There are two forms in which two whorls of stamens can be arranged in Rutaceae: diplostemony and obdiplostemony. According to Kubitzki [1], most genera in Toddalioideae-Rutoideae clade are diplostemonous, such as *Correa*, *Acronychia* and *Choisya*. The ancestral state reconstruction showed the diplostemony evolved several times independently. In the present study, the mature androecium of *Boenninghausenia* was found to be obdiplostemonous, and the ontogenetic pattern shows that obdiplostemony is caused by the shift of the antepetalous stamens. The same situation exists in *Ruta* and *Psilopeganum* [35], and this pattern is thought to be an evolutionarily late phenomenon. As the character state reconstruction showed that obdiplostemony is the ancestral condition in Ruteae (s.s.), it can serve as a morphological synapomorphy for Ruteae and provides good evidence that obdiplostemony was evolved from the haplostemonous androecium.

Polyandry is undoubtedly an advanced character in Rutaceae. The genera of this group that possess polyandrous androecia all belong to Aurantioideae. However, within Aurantioideae, two whorls of stamens also exist in the ancestral genera, e.g. *Bergera*, *Clausena*, *Micromelum*, *Glycosmis*. According to our findings, the development of the androecium of *Murraya exotica* is obdiplostemonous. Based on our observation of the mature flowers, the carpels of *Clausena* and *Glycosmis* are in the antepetalous position. This is a clear indication of obdiplostemony for a pentacyclic flower. Considering the close relationship between these genera and *Murraya*, we assume the developmental mode of androecia is in agreement with *Murraya*. In *Murraya*, the two whorls of stamens arise in a centripetal order, but at later stages, the antepetalous stamens shift externally because of the inflation of the carpellary locules. Current phylogenetic studies showed that *Haplophyllum* is sister to the Aurantioideae clade [39,40]. Based on our observation, the androecium is obdiplostemonous in *Haplophyllum* and the developmental mode is nearly the same with *Murraya*, as well as the genera in Ruteae (s.s.). As *Haplophyllum*, *Murraya*, *Clausena* and *Glycosmis* all possess obdiplostemonous androecia, the ancestral state reconstruction clear shows this is an ancestral character in the Aurantioideae clade.

The varied mode of development of the polyandrous androecium often provides a highly informative systematic character for families and taxa of higher orders. Compared with the polyandry observed in Aurantioideae, obdiplostemony explains the evolutionary mode of the androecium in Aurantioideae. In the floral ontogeny of *Citrus*, a genus with a polyandrous androecium, there is a clear time lag in the initiation of the stamen primordia. The antesepalous stamens are initiated first, and additional stamen primordia are subsequently initiated between these primordia, forming a single whorl [52]. This pattern is reminiscent of the mode of androecium development observed in *Haplophyllum*, *Murraya* and Ruteae (s.s.). The only difference is that instead of the single antepetalous stamen found in those species, there are many stamen primordia at the antepetalous position in *Citrus*. Based on this comparison, we can infer that the numerous stamens observed in Aurantioideae evolved from the obdiplostemonous androecium found in *Murraya*-like genera via increasing the number of antepetalous stamens. Among the Rosids, there are other families that appear to have undergone a secondary increase in their stamen number in the same manner, such as Zygophyllaceae [53]. In *Peganum*, Ronse De Craene reported that antepetalous stamen pairs occur in the obdiplostemonous androecium and interpreted the stamen pairs as a result of a secondary increase. Although in the early stage of the androecium in *Murraya*, antepetalous stamen pairs did not exist, in the floral development of *Haplophyllum*, there are some rare floral buds with antepetalous stamen pairs (Fig 3J). The same situation exists in *Ruta*, a genus in Ruteae (s.s.) clade [35] The antepetalous stamen pairs in *Haplophyllum* and *Ruta* indicate although the odds are low, the situation is quite common in Ruteae-Aurantioideae clade. Why is secondary obdiplostemony often related to an increase in stamen number? The answer is that in this type of obdiplostemony, the antepetalous stamens shift externally because of the maximum use of the floral apex, leaving more space in the antepetalous zone for the increase to occur.

In the current molecular systematics, the genera in traditional tribe Ruteae shows a closer relationship to Aurantioideae than to the traditional subfamily Rutoideae [17]. As the traditionally used fruit type and carpel characters are different between Ruteae and Aurantioideae, obtaining compelling morphological data to support this relationship is important. In the present study, the mode of androecium development was found to be the same in Ruteae (s.s.), *Haplophyllum* and the ancestral genus *Murraya* within Aurantioideae, and the ancestral state reconstruction showed that obdiplostemony can serve as a synapomorphy of the clade. More importantly, the secondary obdiplostemony observed in Ruteae and *Murraya* is both a derived stage from the ancestral haplostemony and a pre-stage for the secondary increase in stamens in Aurantioideae.

Based on our findings, the pattern of androecium development clearly indicates a close relationship between Ruteae and Aurantioideae, and Ruteae is an important clade in the evolution of the androecium in Rutaceae.
